# Effect of Structure on Osteogenesis of Bone Scaffold: Simulation Analysis Based on Mechanobiology and Animal Experiment Verification

**DOI:** 10.3390/bioengineering11111120

**Published:** 2024-11-06

**Authors:** Jialiang Li, Zhongwei Sun, Xinyu Wei, Qinghua Tan, Xijing He

**Affiliations:** 1 Honghui Hospital, Xi’an Jiaotong University, Xi’an 710054, China; 2 Jiangsu Key Laboratory of Engineering Mechanics, School of Civil Engineering, Southeast University, Nanjing 210096, China; zw-s@seu.edu.cn; 3 Department of Health Management, The Second Affiliated Hospital of Xi’an Jiaotong University, Xi’an 710014, China; xinyu_wei0721@126.com; 4 Department of Orthopedics, The Second Affiliated Hospital of Xi’an Jiaotong University, Xi’an 710014, China; tanqinghua33@126.com (Q.T.); xijing_h@vip.tom.com (X.H.)

**Keywords:** scaffolds, computational simulation, mechanobiology, tissue regeneration, osteogenesis, animal experiment

## Abstract

Porous scaffolds, whose mechanical and biological properties are greatly affected by structure, are new treatments for bone defects. Since bone repair is related to biomechanics, analyzing the osteogenesis in scaffolds based on mechanical stimulation may become a more effective method than traditional biological experiments. A tissue regeneration algorithm based on mechanical regulation theory was implemented in this study to evaluate the osteogenesis of classical scaffolds (Gyroid, I-WP, and Diamond). In vivo experiments were used to verify and supplement the simulation results. Different approaches to describing osteogenesis were discussed. Bone formation was more obvious inside the Gyroid scaffold and outside the I-WP scaffold, while the new bone was more sufficient and evenly distributed in the Diamond scaffold. The osteogenesis pattern of the bone scaffold in the simulation analysis was consistent with the results of animal experiments, and the bone volume calculated by the tissue fraction threshold method and the elastic modulus threshold method was very similar to the in vivo experiment. The mechanical responses mediated by structure affect the osteogenesis of bone scaffolds. This study provided and confirmed a simulation analysis method based on mechanical regulation theory, which is more efficient and economical for analyzing tissue healing in bioengineering.

## 1. Introduction

Bone disorders, including osteoarthritis, fractures, and skeletal malformations, can cause bone defects, often requiring surgical treatment [1]. A bone implant with sufficient mechanical strength can be used to reconstruct the bone structure [2]. In recent years, researchers have concentrated more on bone implants’ osteogenic capacities and function [3]. The porous structure is essential for bone implants as it can provide the space for the ingrowth of cells and tissues and the penetration of blood vessels, thus leading to bone formation and eventually enhancing the long-term stability of implants [4]. Therefore, further exploration of the design and properties of porous structures (porous scaffolds) will hopefully benefit the clinical application of porous bone implants.

Numerous methods are available for designing porous structures, such as computer-aided design, parametric approaches, and imaging methods [5]. Among these, the Diamond porous structure is widely used because of its excellent mechanical strength (its bearing capacity is the highest in the previous study), which is important for the implants to restore the stability of the bone defects area [6,7]. Also, the Gyroid structure with outstanding fluid responsiveness has drawn extensive attention [8]. Studies on the optimization of the curved surface structure mainly focus on the improvement of the flow pattern of the fluid in the scaffold because the resulting hydromechanical stimulation may be related to the osteogenic signal [9,10]. However, porous structures with different morphologies have a complex impact on biomechanical response and osteogenesis, which need to be further explored by comprehensive studies [11].

Most of the traditional studies prepared porous scaffolds first and then evaluated the osteogenic properties of scaffolds through a series of in vitro and in vivo experiments [12,13]. These methods are intuitive and persuasive; however, they take lots of time and effort and cannot detect a large sample of different types of porous scaffolds. Considering the osteogenic differentiation of mesenchymal stem cells (MSCs) relates to the biomechanical characteristics [14], which can be detected by computer simulation, some computer-assisted predictive models based on mechanical stimuli can be used to detect osteogenesis. Previous studies proposed a binomial finite element (FE) method; however, this approach ignores the dynamic changes in cell differentiation [15]. Also, some bone-remodeling algorithms were further improved and optimized to achieve dynamic simulation and even cell migration [16]. With the development of biology and computer science, bionic simulations have become a cost-effective way to analyze osteogenesis [17]. However, the accuracy and reliability of these methods need further validation.

This study combined the mechanical stimuli simulation method and mechano-regulation theory to predict the osteogenic capacities of classical porous structures. Meanwhile, the data from the simulation and the information visualization (definition of osteogenesis) were discussed. Also, an in vivo experiment was implemented to estimate and validate the simulation results. The proposed simulation method can potentially become an effective, time-saving, and economical approach for analyzing a porous structure and tissue healing.

## 2. Materials and Methods

### 2.1. Modeling of the Different Unit Cell Geometries

**Figure 1:**
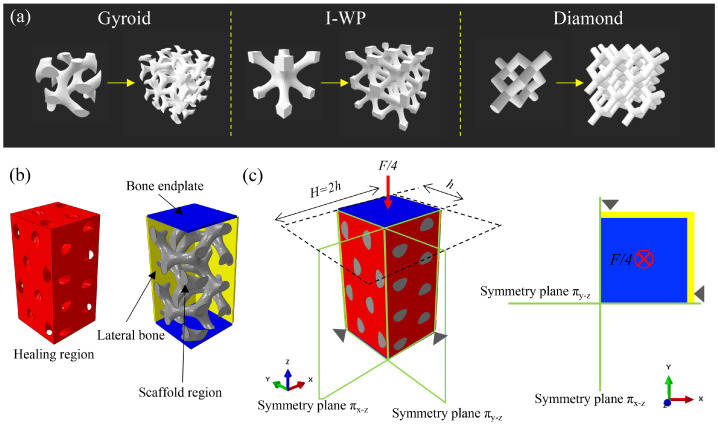
Design of bone scaffolds and the establishment simulation analysis model. (**a**) The unit cell and porous scaffold of Gyroid, I-WP, and Diamond structure. (**b**) Simulation analysis model. Exploiting the symmetry of the problem, the volume analyzed was reduced to one-quarter of the total volume. The model consisted of four regions, namely, the healing region (red), the scaffold region (grey), the bone endplate (blue), and the lateral bone (yellow). The regions are connected through node sharing at the interface. (**c**) Boundary and loading conditions acting on the simulation model.

Three classical reticular porous structures were designed and applied in the present study, including Diamond, Gyroid, and I-WP structures. The implicit surface method was employed to design the unit cells (Figure 1a); the related formulas are as follows:

Diamond [18]:

*F*(*x*,*y*,*z*) = sin(*x*)sin(*y*)sin(*z*) + sin(*x*)cos(*y*)cos(*z*) + cos(*x*)sin(*y*)cos(*z*) + cos(*x*)cos(*y*)sin(*z*) = 0, (1)

Gyroid [19]:

*F*(*x*,*y*,*z*) = cos(*x*)sin(*y*) + cos(*y*)sin(*z*) + con(*z*)sin(*x*) − c = 0, (2)

I-WP [20]:

*F*(*x*,*y*,*z*) = 2[cos(*2πx*)cos(*2πy*) + cos(*2πy*)cos(*2πz*) + cos(*2πx*)cos(*2πz*)] − [cos(*4πx*) + cos(*4πy*) + cos(*4πz*)] = 0, (3)

The parametric modeling of the different cells was performed by writing Python scripts that were given as input to ABAQUS 2017 (Dassault Systèmes, Vélizy-Villacoublay, France). When the topology of the porous structure was fixed, porosity, pore size, and size of a single-unit cell were a set of three dependent parameters. In this research, the size of different unit cells and porosity were set as cubic with the side long h = 1560 μm and 80%, respectively. Such generated unit cells were replicated by correctly mirroring them. The resulting porous scaffold model had a cubic shape with two layers of unit cells in each direction (the sidelong H = 2 × h = 3120 μ − m)

### 2.2. Modeling of the Scaffold and Granulation Tissue System

Poroelastic finite element models of Gyroid, Diamond, and I-WP scaffolds were generated. In exploiting the symmetry of the porous model, the volume analyzed was reduced to one-quarter of the total volume to reduce the computational cost. The process of osteogenic differentiation of MSCs is extremely important for bone formation. Hence, we assumed that well-vascularized granulation tissue had already occupied the internal space of scaffolds in the initial configuration (namely healing region), which was obtained with the Boolean operation (highlighted in red, Figure 1b). A tie constraint was established between the surfaces of the scaffold and those adjacent to the granulation tissue.

Considering that the surrounding bone tissue affects osteogenesis after scaffold implantation, bone endplate models were generated on the scaffold model’s top, bottom, and lateral surfaces. Two reference points were generated at the upper and lower surface of the bone endplate. A compression load was applied on the upper reference point, and load F was hypothesized according to the superior surface area of the scaffold and a force per unit area (*p* = 0.5 MPa) (Figure 1c). The bottom reference point of the model was established by fixing all the degrees of freedom.

The material properties of the different tissue types are given in Table 1 [16]. Lateral bone endplates were generated solely to provide bone growth conditions and thus were assigned a small elastic modulus. The tetra-mesh algorithm was utilized to mesh the entire model.

### 2.3. Tissue Regeneration Algorithm

Bone regeneration includes cell differentiation, tissue destruction, intramembranous ossification, endochondral ossification, and tissue resorption [21]. Osteogenesis, chondrogenesis, fibrous tissue development, and bone remodeling were observed to occur consistently, which was achieved by cell differentiation. Precursor cells (MSCs) were allowed to differentiate into fibroblasts, chondrocytes, or osteoblasts according to different mechanical environments. Based on the mechanical stimulations and a set of additional conditions (Table 2) [22,23,24], we performed MSC differentiation, where different cell types were represented by their respective tissue phenotypes.

**Figure 2:**
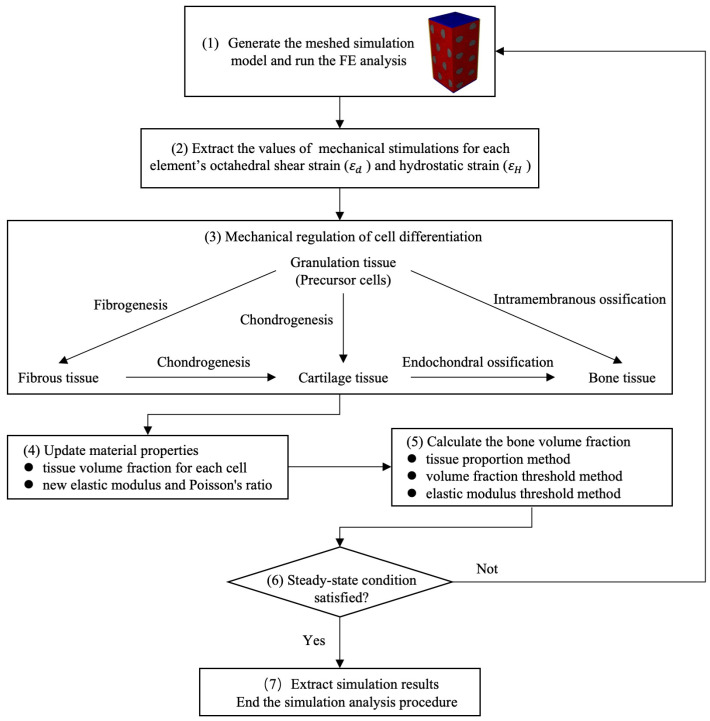
Schematic of the algorithm to determine the tissue regeneration processes. Firstly, simulation analysis models based on different structures were established and run (1). Then the octahedral shear strain ($\varepsilon_{d}$) and hydrostatic strain ($\varepsilon_{H}$) of each element were extracted (2), and the differentiation direction of the internal tissue of the model was determined according to the mechanical regulation theory (3). The material properties of each component in the model were updated according to the differentiation results (4). The proportion of different tissue in each iteration was calculated (5). Finally, analysis was stopped (6) and results were extracted (7) until the simulation system reached stability.

The regeneration algorithm process is shown in Figure 2. Mechanical stimulation values included octahedral shear strain ($\varepsilon_{d}$) and hydrostatic strain ($\varepsilon_{H}$), which were calculated according to Equations (4) and (5)

(4) $$\varepsilon_{d}=\frac{2}{3}\sqrt{{\left(\varepsilon_{1}-\varepsilon_{2}\right)}^{2}+{\left(\varepsilon_{2}-\varepsilon_{3}\right)}^{2}+{\left(\varepsilon_{1}-\varepsilon_{3}\right)}^{2}}$$

(5) $$\varepsilon_{H}=\frac{1}{3}(\varepsilon_{1}+\varepsilon_{2}+\varepsilon_{3})$$

where $\varepsilon_{1}$, $\varepsilon_{2}$ and $\varepsilon_{3}$ are principal strains.

In each iteration, cell differentiation and tissue growth were first evaluated, after which the new material properties (elastic modulus and Poisson’s ratio) of each element were recalculated based on the updated tissue composition (according to Equations (6)–(8)) as follows:

(6) $$E=E_{gran}m_{gran}^{3}+E_{fib}m_{fib}^{3}+E_{cart}m_{cart}^{3}+E_{bone}m_{bone}^{3}$$

(7) $$v=v_{gran}m_{gran}+v_{fib}m_{fib}+v_{cart}m_{cart}+v_{bone}m_{bone}$$

Qualifiers:

(8) $$m_{gran}+m_{fib}+m_{cart}+m_{bone}=1$$

where E is the Young’s modulus, v is the Poisson’s ratio, and $m_{gran}$, $m_{fib}$, $m_{cart}$, and $m_{bone}$ are the volumetric fractions of granular tissue, fibrous tissue, cartilage, and bone, respectively.

The simulations stopped running when the bone volume fraction reached a steady state, according to Equation (9)

(9) $$\frac{{vf}_{bone}^{10\left(i+1\right)}-{vf}_{bone}^{10i}}{{vf}_{bone}^{10\left(i+1\right)}}<0.05$$

where ${vf}_{bone}^{10i}$ is the bone volume fraction after being calculated at iteration 10i.

### 2.4. Three Approaches to the Description of Bone Volume Fraction

As there were inevitable differences between the simulation results and the in vivo experiment, we proposed three calculation approaches for describing bone volume fraction, including the tissue proportion method, the volume fraction threshold method, and the elastic modulus threshold method.

In the tissue proportion method, bone volume fraction was defined as the ratio of bone volume to the initial granulation tissue volume (the volume of the healing region), as in Equation (10):

(10) $$vf_{bone}=\frac{\sum_{i=0}^{N}v_{ele}^{i}m_{bone}^{i}}{\sum_{i=1}^{N}v_{ele}^{i}}$$

where *N* is the total number of elements in the healing region, $v_{ele}^{i}$ is the volume of element i, and $m_{bone}^{i}$ is the bone volume fraction of element i.

In the tissue fraction threshold method, when the bone tissue fraction within an element is ≥0.25, the whole element is regarded as bone tissue, i.e., the bone volume fraction is 1. The tissue proportion method calculated the bone volume fraction of the remaining elements, as in Equations (11) and (12):

(11) $$v_{bone}=\sum_{j=1}^{M}v_{ele}^{j}+\sum_{i=1}^{N}v_{ele}^{i}m_{bone}^{i}$$

(12) $$vf_{bone}=\frac{v_{bone}}{\sum_{i=1}^{N+M}v_{ele}^{i}}$$

where *M* is the total number of elements satisfying the bone volume fraction ≥ 0.25, *N* is the total number of elements satisfying the bone volume fraction of <0.25, $v_{ele}^{i}$ is the volume of element i, and $m_{bone}^{i}$ is the bone volume fraction of element i.

In the elastic modulus threshold method, an element is considered to have a bone volume fraction of 1 when its elastic modulus is ≥10 MPa. The tissue proportion method calculated the bone volume fraction of the remaining elements, as in Equations (13) and (14):

(13) $$v_{bone}=\sum_{j=1}^{A}v_{ele}^{j}+\sum_{i=1}^{B}v_{ele}^{i}m_{bone}^{i}$$

(14) $$vf_{bone}=\frac{v_{bone}}{\sum_{i=1}^{A+B}v_{ele}^{i}}$$

where *A* is the total number of elements satisfying the elastic modulus is ≥10 MPa, *B* is the total number of elements satisfying the elastic modulus of <10 MPa, $v_{ele}^{i}$ is the volume of element i, and $m_{bone}^{i}$ is the bone volume fraction of element i.

### 2.5. In Vivo Animal Experiments

#### 2.5.1. Surgical Procedure

All animal procedures and experiments were approved by the Ethics Committee of Xi’an Jiao Tong University and were performed in compliance with the Regulations of the Administration of Affairs Concerning Experimental Animals of China. A total of 6 adult male New Zealand rabbits (3–3.5 kg), obtained from the Animal Experiment Center of Xi’an Jiao Tong University, were used for this experiment. Twelve femurs of 6 rabbits were randomly assigned to 3 types of scaffolds (Φ = 4, h = 6 mm) (*n* = 4). Porous scaffolds based on these three structures (Gyroid, Diamond, and I-WP) were prepared from Ti–6Al–4V alloy and manufactured by the selective laser melting 3D printing system BLT-S200 (Bright Laser Technologies, Xi’an, China). Briefly, Ti–6Al–4V powders were spread in a powder bed by a scraper, then laser scanning was carried out layer by layer according to the computer model, and, finally, the remaining powders were removed to retain the scaffold. After 3D printing, all scaffolds were heat-treated at 800 °C for 2 h in an argon gas atmosphere, followed by ultrasonic cleaning with ethanol and distilled water successively.

**Figure 3:**
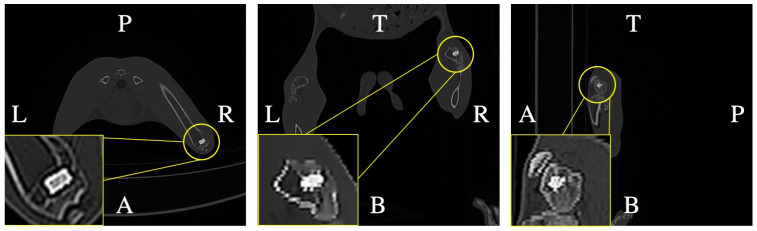
Postoperative computed tomography. The scaffold position was defined by postoperative computed tomography examination. The yellow frame shows a larger picture of the scaffold inside the femur.

The operation procedure was the same as previously reported [25]. After disinfection and anesthesia, the skin and fascia were incised, followed by exposure of the distal femur. Next, the scaffold was implanted after drilling the bone defect area, and, finally, the surgical incisions were sutured in layers. Postoperative CT (HUADONG electronic, Yantai, China) (Figure 3) was used to observe the position of the scaffolds. Femoral samples were collected 4 weeks after the operation and fixed in 4% paraformaldehyde for subsequent studies.

#### 2.5.2. Micro-CT Analysis and Histological Evaluation

Micro-CT was used to observe the osteogenesis and to quantitatively analyze the newly formed bone (NF bone). The region of interest was set as the 3D cylindrical space bounded by the edge of the scaffold. Bone volume fraction was the ratio of bone volume to the entire 3D space except for the scaffold volume, reflecting the amount of NF bone. The sections were stained with 1.2% trinitrophenol and 1% acid fuchsin (VG staining), after which they were observed under an optical microscope (Olympus Corporation, Tokyo, Japan).

### 2.6. Statistical Analysis

The data of bone volume/total volume (BV/TV) were expressed as mean ± standard deviation and analyzed using SPSS (version 19.0, SPSS Inc., Chicago, IL, USA). A one-way analysis of variance (ANOVA), followed by an LSD (least significant difference) test, was used to compare BV/TV among the three treatment groups. *p*-values < 0.05 were considered statistically significant.

## 3. Results

### 3.1. Effect of Manner of Calculation on the Tissue Volume Fraction

**Figure 4:**
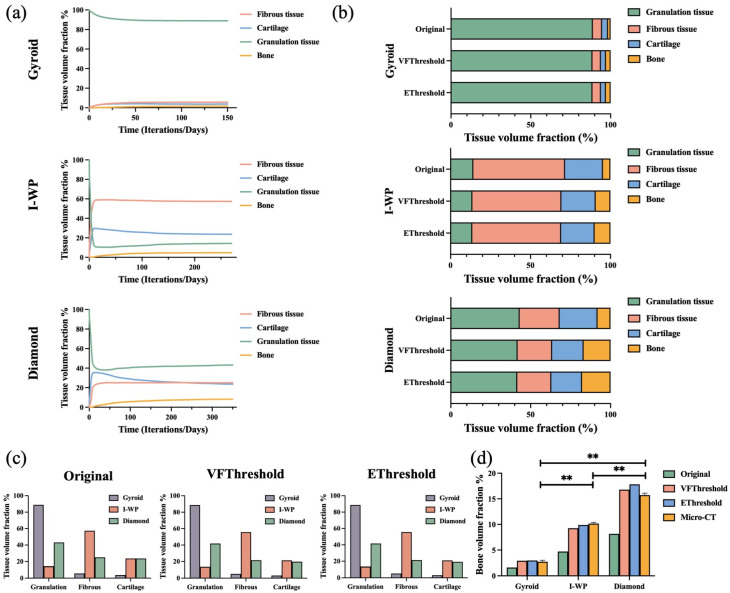
(**a**) Temporal evolution of tissue fraction in scaffolds. (**b**) Tissue fraction calculated by the tissue proportion method (Original), volume fraction threshold method (VFThreshold), and elastic modulus threshold method (EThreshold). (**c**) Comparison of the volume fraction of granulation tissue, fibrous tissue, and cartilage tissue in different scaffolds. (**d**) Comparison of bone volume fraction in simulation analysis of different scaffolds with animal experiment results. *p* ** < 0.01.

The differentiation phase of MSCs initiated the tissue regeneration process, where differentiated cells represented their respective tissue phenotypes. Progressively, bone, cartilage, and fibrous tissue grew in different proportions and locations on scaffolds. After at least 150 iterations, the volume fraction of different tissue had leveled off (Figure 4a).

Due to the consistency of the number of elements in the same scaffold model, the tissue volume fraction reflected the growth of different tissues. As illustrated in Figure 4b, the sum of the tissue volume fraction is 100%, and the results of three methods of describing tissue ratio are compared. The tissue proportion method is to directly count the proportion of elements, while the tissue fraction threshold method and the elastic modulus threshold method are employed to determine the element as a complete bone after it reaches a certain bone tissue proportion (≥0.25) or elastic modulus (≥10 MPa). For all the scaffolds, the tissue volume fractions of granulation tissue, fibrous tissue, and cartilage calculated using the tissue proportion method were all greater than those calculated using the tissue fraction threshold method and the elastic modulus threshold method. For example, in the Diamond scaffold, the tissue volume fractions of fibrous tissue calculated by the tissue proportion method, the tissue fraction threshold method, and the elastic modulus threshold method were 25.07%, 21.58%, and 21.34%, respectively. Conversely, bone volume fraction was the greatest in the elastic modulus threshold method, i.e., it was twice that calculated by the tissue proportion method.

### 3.2. Comparison of Tissue Volume Fraction in Different Scaffolds

The volume fraction of each tissue was not identical in different scaffolds. Also, the proportional distribution of different tissues had a similar performance according to different calculation methods (Figure 4c). In the Gyroid scaffold, for example, calculated by the elastic modulus threshold method, granulation tissue occupied the vast majority (88.68%), and its volume was two copies of that in the Diamond (41.64%) and almost six times that in the I-WP (14.27%). Moreover, the formation of fibrous tissue was much more evident in the I-WP model than in the other two models. The situation was similar for cartilage growth in the I-WP and Diamond models, but the volume fraction of both was much higher than the Gyroid model.

The bone volume fraction was acquired by the simulation model and by scanning animal specimens, respectively. As shown in Figure 4d, for micro-CT results, the volume of NF bone in the Gyroid scaffold (2.71 ± 0.30%) was less than that in the I-WP scaffold (10.15 ± 0.26%), while the Diamond scaffold had more NF bone (15.72 ± 0.39%). At the same time, the bone volume fraction, which was calculated using the tissue fraction threshold method (2.91% in Gyroid, 9.26% in I-WP, and 16.78% in Diamond) or the elastic modulus threshold method (2.96% in Gyroid, 9.89% in I-WP, and 17.83% in Diamond), closely approximated that in micro-CT scanning and was significantly higher than that in the tissue proportion method (1.62% in Gyroid, 4.71% in I-WP, and 8.15% in Diamond). This difference means that the results of the simulation analysis cannot be directly propagated. Thus, optimizing the calculation methods according to osteogenesis characteristics is of vital importance.

### 3.3. Visualized Bone Formation

#### 3.3.1. Bone Formation in Simulation

**Figure 5:**
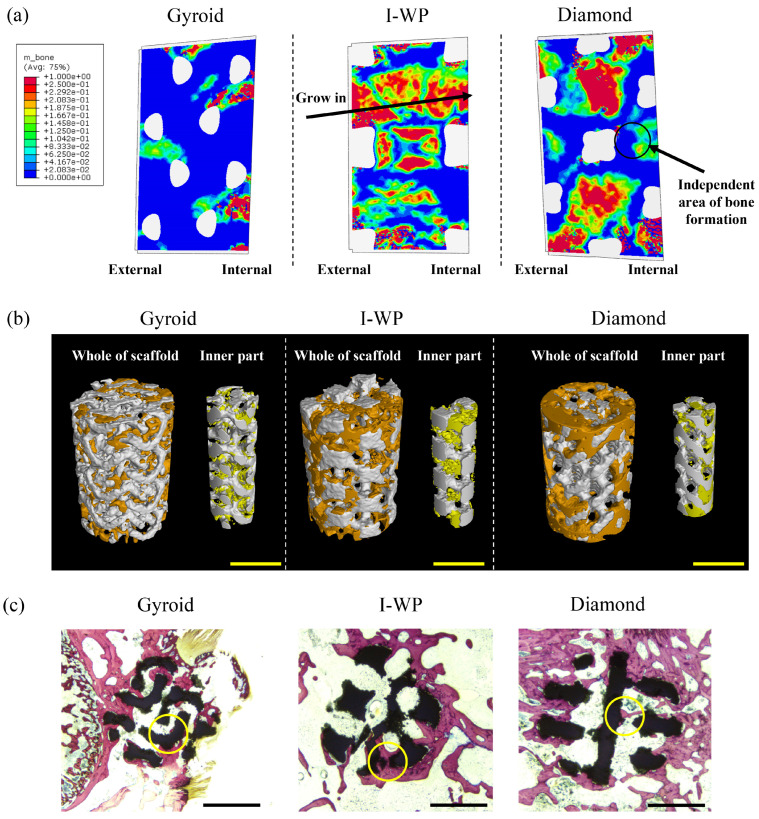
(**a**,**b**) Visualization of osteogenesis forms within the different scaffolds in simulation analysis and in Micro-CT. Bar: 2 mm. (**c**) The observation of VG staining. Bone: red; scaffold: black; cell nucleus: blue. Optical microscope bar: 2 mm.

After the simulation system was stabilized, the proportion and location of NF bone were visualized (Figure 5a). Taken as a whole, the bone formation was even more pronounced in the I-WP and Diamond models than in the Gyroid model. Bone growth started from the peripheral bone endplate and the interior, respectively. In the Gyroid model, some bone growth was seen in the lateral portion, and, more importantly, new bone formed mainly inside the Gyroid scaffold. In contrast, in the I-WP model, a large amount of NF bone formed where the scaffold was in contact with the bone endplate and gradually grew into the scaffold based on this. However, there was no obvious independent area of NF bone formation in the inner region of the I-WP scaffold. In contrast, the growth of NF bone in the Diamond scaffold was more balanced and pronounced, and independent bone formation areas were also observed inside the scaffold.

#### 3.3.2. Bone Formation Analyzed by Micro-CT

In order to test the reliability of the simulation analysis on the bone formation pattern, micro-CT scanning was performed to obtain the bone growth inside the scaffold. At the same time, we observed the whole and the inner part of the scaffold separately (Figure 5b). In the Micro-CT reconstruction model, the new bone formation was scattered on the surface of the Gyroid scaffold, and the NF bone was relatively obvious inside the scaffold. The abundant new bone could be seen in the area where the I-WP scaffold contacted the surrounding bone tissue. Similar to the simulation analysis, significant new bone formation was observed inside and around the Diamond scaffold.

#### 3.3.3. Bone Formation Analyzed by Tissue Staining

Bone tissue staining could more directly observe the growth of NF bone in the injured area. In VG staining (Figure 5c), it was clear that all the original surrounding bone invaded and grew into the scaffold and gradually wrapped the scaffold. A few independent areas of NF bone were observed inside the Gyroid scaffold, and the outer region of the I-WP scaffold was more fully filled with NF bone. Notably, the growth of NF bone in the Diamond scaffold was more remarkable, and a bone bridge even appeared to connect the wall of the pore.

## 4. Discussion

The porous scaffold is an effective therapy for bone disorders, and its structural morphology and parameters are vital for bone formation and ingrowth. Currently, there is a set of well-established assessment methods for the osteogenic capability of scaffolds, including characterization analysis and in vitro and in vivo experiments [26,27]. However, this assessment system has a heavy workload and often only studies the phenomenon of osteogenesis but ignores the underlying mechanism. Therefore, the intention of this study is to simulate the mechanical mechanism of osteogenesis and develop a rapid analytical method which will have practical significance for the future application of bone implants.

Our previous study revealed that the structure or parameters of the bone scaffold affected the bone formation because of different hydrodynamics and the flow-through format [25]. The differentiation of precursor cells into mature osteoblasts was correlated with a mechanical stimulus. From this perspective, establishing biomimetic models based on mechanical stimulation is a feasible method to analyze the osteogenesis. Rodríguez-Montaño has combined the biophysical stimuli (such as octahedral shear strain) and FE analysis method to determine tissue formation in the scaffold [15]. This was a good attempt to predict the biological properties of the bone scaffold; however, this method only considers the alteration of a single mechanical factor. Calvo-Echenique further introduced bone mineral density parameters into the analysis for mediation and added indicators of mechanical stimulation [22], thus improving the reliability of the simulation. Accordingly, the octahedral shear and hydrostatic strain were integrated into the present study to evaluate tissue differentiation, and the analysis of mechanical signals was more comprehensive [28,29]. Meanwhile, iterative computation was also used to simulate the dynamic changes of osteogenesis. In the simulation model, granulation tissue was set to uniformly fill the entire scaffold, which is a departure from reality [30]. Therefore, some scholars argued that the dynamic diffusion of cells should also be considered [22]. Although this method still needs to be optimized, it also provides an improved direction for subsequent analysis. From this point of view, this study combines iterative methods with biomechanics to develop a more economical and reliable analysis method based on previous studies.

Although simulation analysis has gradually become a useful means for predicting the performance of scaffolds, the reliability of the results still needs to be verified. Therefore, animal experiments are essential as they may provide robust verification of the simulation analysis results of this study. However, although animal experiments are more descriptive, conventional detection methods are one-sided [31]. For example, due to the low gray level of the new bone, it is difficult to distinguish it from the original bone, which affects micro-CT analysis. In addition, the location and staining method of tissue sections can also affect the histochemical analysis. In the computational simulation, the volume and tissue proportion of all units are uniquely determined, which helps to avoid the problematic differentiation of bone tissues in animal experiments. However, there is still a gap between the computer model and the real body environment. Since cartilage and bone tissue are anisotropic materials, linear elastic models can be used to simulate the biological activity of tissues, but they still have limitations, so more detailed and accurate analytical models are necessary. Therefore, the validated simulation analysis method may be a more reliable evaluation method.

It is important to note that osteogenesis is a dynamic process in which the extracellular matrix gradually mineralizes and maturates rather than suddenly changing from granulation tissue or cartilage to bone [32]. Therefore, the criteria for skeletal maturation also need to be seriously discussed in simulation analysis; e.g., when 20% of a unit is calculated as bone tissue, it may already be mature bone. Therefore, the description of simulation results is also important. This study proposes three methods to describe tissue ratio and innovatively combines simulation results with animal experiments to provide a reference for the data processing of simulation analysis. The tissue proportion method is more intuitive but may differ from the real situation. The elastic modulus threshold method considers the difference in mechanical properties between bone and surrounding tissue [33]. In the tissue fraction threshold method, when the volume of bone tissue is larger than a certain percentage, it can be considered that the unit is undergoing skeletal maturation. Therefore, the three description methods need to be compared and analyzed.

In this study, the proportion of each tissue was gradually balanced with the increase in the number of iterations. In the same scaffold, the tendency of the three description modalities to describe tissue proportions was consistent; e.g., in the Gyroid scaffold, granulation tissue was the most abundant, followed by fibrous tissue and cartilage tissue, and bone tissue was the scarcest in all three descriptions. However, the optimized description of bone tissue showed that the proportion of bone tissue in the elastic modulus and tissue fraction threshold methods was much higher than that of the tissue proportion method. In addition, the results of both methods were similar to those of micro-CT scanning in animal experiments, thus suggesting that these two methods can provide rather real descriptions. The main function of bone is the transmission of mechanical signals, so when the mechanical properties of an element have reached the mechanical properties similar to that of mature bone with the increase in iterations, it can be considered as mature bone, without the need for the entire element to become mature bone. Therefore, this study proposes to process the simulation results by the elastic modulus and tissue fraction threshold methods and has obtained credible evidence from animal experiments. However, the definition of mature bone still needs to be further explored.

In addition to quantitative analysis, simulation analysis can more intuitively and qualitatively predict the pattern of NF bone in scaffold [34,35]. Due to the diamond-like appearance of the single unit cell, the Diamond scaffold has a strong mechanical bearing capacity [36]; Gyroid and I-WP scaffolds have a curved surface structure, which is very conducive to fluid interaction [37]. However, how the three classical structures affect bone formation needs to be further elucidated. In the present study, we found that the bone formation of the Gyroid scaffold was not obvious, while there was still more granulation tissue. In addition, the site of NF bone in the Gyroid scaffold was closer to the inside of the scaffold. The I-WP scaffold had more obvious cartilage organization, and the NF bone was closer to the periphery of the scaffold. The NF bone tissue of the Diamond scaffold was more obvious and more evenly distributed. More importantly, the results of the animal experiments were consistent with those of the simulation analysis. Micro-CT and histochemical analyses (VG staining) confirmed that the Diamond scaffold had more adequate bone formation, while the inner part of the Gyroid scaffold and the outer part of the I-WP scaffold had more obvious bone formation.

Previous studies focused on the amount of new bone formation but ignored the location and form of bone formation [38]. Simulation analysis can help better understand and predict a structure’s influence on osteogenesis. We can speculate that the different hydrodynamic characteristics lead to the different cell distribution and osteogenesis patterns in different structures [10,39]. However, previous studies on the structure of the Gyroid scaffold have also reported different results on its osteogenic behavior [40]. This may be because structures may have different osteogenic performances under different mechanical environments. In addition, bone defects may require unique mechanical characteristics of the scaffold, such as the carrying capacity of the scaffold in the load-bearing area. Therefore, there may be distinct structural choices in the face of different mechanical environments. It is worth mentioning that simulation analysis is an effective and economical method to study the influence of different mechanical bearing environments on the performance of scaffolds.

Simulation analysis can be used to study and compare multiple factors of osteogenesis at the same time, thus being more time-saving than traditional biological experimental methods. However, in addition to biomechanical factors, bone scaffold materials and the in vivo environment greatly impact osteogenesis [41,42], which may cause differences in simulation analysis results. Moreover, it is urgent to develop analytical models that resemble the characteristics of real organizations. Still, it is undeniable that the analysis results have an important predictive effect on the research of bone scaffolds. It is worth noting that this simulation analysis is based on mechanical regulation theory, so combining the simulation experiment with the mechanical stimulation pathway of an osteoblast cell line may become a new method to explore the cytological mechanism of osteogenesis.

## 5. Conclusions

In this study, we successfully established an iterative simulation analysis method to evaluate the osteogenic performance of bone scaffolds based on mechanical stimulation, after which the analysis results were verified by animal experiments. At the same time, we discussed different methods of description for osteogenic results. Our results showed that the amount and form of the bone formation of the bone scaffolds with different structural designs were diverse and the elastic modulus threshold and tissue fraction threshold methods were closer to the animal experiments in describing the bone formation. Therefore, this method can be used to more conveniently and reliably predict and analyze the osteogenic performance of bone scaffolds, having a good application prospect.

## Figures and Tables

**Table 1:** Material properties utilized in the simulation model.

Material	Yong Modulus (MPa)	Poisson’s Ratio
Bone endplates	10,000	0.325
Scaffold	1000	0.2
Side bone	0.01	0.325
Granular tissue	0.2 [16]	0.167 [16]
Fibrous tissue	2 [16]	0.167 [16]
Cartilage	10 [16]	0.167 [16]
New bone	1000	0.325

**Table 2:** Material properties utilized in the simulation model [22,23,24].

Event	Stimuli	Additional Rules	Rates
Tissue destruction	*ε_H_* > 5% OR *ε_d_* > 15%	N.A.	Return to granular tissue
Fibrous tissue formation	(−1% < *ε_h_ <* 5% AND 5% < *ε_d_* < 15%) OR (1% < *ε_h_*< 5% AND *ε_d_* < 15%)	N.A.	0.2
Cartilage tissue formation	(−5% < *ε_h_* < −1% AND 5% < *ε_d_* < 15%)	(Cartilage fraction < 25% AND bone fraction < 75%)	0.1
Endochondral ossification	(−5% < *ε_h_* < −0.1% AND *ε_d_* < 5%)	Tissue vascularized AND bone fraction in neighbouring elements > 25% AND cartilage fraction > 25%	0.1 (Based on the fraction of cartilage bone)
Intramembranous ossification	(−0.1% < *ε_h_* < 1% AND 1% < *ε_d_* < 5%) OR (0.1% < *ε_h_* < 1% AND *ε_d_* < 1%)	Tissue vascularized AND bone fraction in neighbouring elements > 25% AND cartilage fraction < 25%	0.1 (Precursor cell to osteoblasts)
Tissue resorption	(−0.1% < *ε_h_* < 0.1% AND *ε_d_* < 1%)	N.A.	0.05 (Based on the fraction of bone)

## Data Availability

The data presented in this study are available on request from the corresponding author due to the data still being analyzed in unpublished studies.
